# Chemoresistive Sensor Readout Circuit Design for Detecting Gases with Slow Response Time Characteristics

**DOI:** 10.3390/s22031102

**Published:** 2022-01-31

**Authors:** Dong-Yeon Lee, Joon-Boo Yu, Hyung-Gi Byun, Hyeon-June Kim

**Affiliations:** Department of Electronics Engineering, Kangwon National University, Samcheok 25913, Korea; ldylee5423@kangwon.ac.kr (D.-Y.L.); joonbou.yu@kangwon.ac.kr (J.-B.Y.)

**Keywords:** gas sensor, chemoresistive sensor, slow response gas sensing, readout circuit, offset tracking readout

## Abstract

Based on an analysis of the signal characteristics of gas sensors, this work presents a chemoresistive sensor readout circuit design for detecting gases with slow response time characteristics. The proposed readout circuit directly generates a reference voltage corresponding to the initial value of the gas sensor and extracts only the amount of gas concentration change in the sensor. Because the proposed readout circuit can adaptively regenerate the suitable reference voltage under various changing ambient conditions, it can alleviate the variation in output values at the same gas concentration caused by non-uniformities among gas sensors. Furthermore, this readout circuit effectively eliminates the initial value shifts due to the poor reproducibility of the gas sensor itself without requiring complex digital signal calibrations. This work focuses on a commercially viable readout circuit structure that can effectively obtain slow response gas information without requiring a large capacitor. The proposed readout circuit operation was verified by simulations using spectre in cadence simulation software. It was then implemented on a printed circuit board with discrete components to confirm the effectiveness with existing gas sensor systems and its commercial viability.

## 1. Introduction

Recently, with the development of electronic devices, the demand for microsensor applications has been increased for improving the quality of human life. In particular, gas sensors such as electronic noses (e-noses) are being widely used in various electronic application fields, such as air-quality monitoring systems, hazardous gas detection systems, and healthcare systems [1,2,3,4]. To date, various types of gas sensors have been developed based on different sensing materials and their specialized sensing mechanisms [5,6,7]. Recently, metal-oxide-semiconductor (MOS)-based gas sensors are attracting attention with the development of semiconductor manufacturing technology owing to their compatibility with Internet of Things (IoT) applications. In addition, these sensors have chemoresistive characteristics and provide a simple interface circuitry for the signal conversion.

When a gas sensor is exposed to the target ambient conditions, depending on the sensing materials, their chemoresistance exhibits various ohmic distributions and different sensitivity and selectivity. The purpose of e-nose is to detect the type of target gas using the various sensing materials. From a signal and system’s perspective, if the type of target gas can be clearly defined, the sensor interfaces and readout integrated circuits (ROICs) can be efficiently designed for the optimal signal extraction. However, due to inherent properties of the materials constituting the gas sensor (i.e., poor selectivity of gas sensors [8,9]), it is difficult to rely on the selectivity of gas sensors that should react only to a specific gas component. Moreover, the chemoresistive characteristics of gas sensors change in accordance with the surrounding conditions in the environment, such as temperature, humidity, and pressure [10]. This leads to different initial values of the gas sensor (i.e., offset variations) for each set of sensing measurements, causing non-uniformity of signal conversion results even with the same gas concentration. To alleviate this problem, because there is a limitation for improving gas sensor performance only through the physical structure and material modifications, the studies on gas sensing ROICs should be performed together with the sensor characteristics to effectively overcome the limits of gas sensor performances in this field.

As a conventional readout topology, most commercial chemoresistive gas sensors are based on a voltage-divider structure [11] for resistance-to-voltage conversion because it provides a wide dynamic range of the sensor resistance. In addition, to remove the initial value of gas sensors (i.e., gas sensor offsets), a differential amplifier and Wheatstone bridge circuit [12,13,14] are utilized to obtain only the amount of change of the gas sensor output with a fixed proper reference voltage. However, since the gas sensor has an offset variation for each sample, gas sensor offset cancelling circuits with a fixed reference voltage do not accurately extract the amount of change of the gas sensor output, resulting in a degradation of uniformity and dynamic range. This phenomenon is further exacerbated when the gas sensor offset is changed by the surrounding conditions in the environment.

Until now, various types of sensing methods have been reported: the oscillator-based sensing method [15] to perform low-power conversion, the digital-to-analog converter (DAC)-based method [16] to support wide-range resistance change, and the reconfigurable multiple sensing method [17] to detect various kinds of gases with different sensitivity and resistance characteristics. With the same motivation as previous works [18,19], based on the analysis of the electrical signals of the gas sensor, this paper presents a slow-response gas-sensing readout circuit that minimizes the effect on sensor offset variation caused by various ambient conditions. After reading out a gas sensor offset, using an additional digital to analog convertor (DAC), the proposed readout circuit generates a suitable reference voltage for a differential amplifier. Then, while maintaining the uniformity for each gas sensor, the differential amplifier extracts only the amount of change in the gas sensor. Furthermore, because the proposed readout circuit adaptively generates the suitable reference voltage, the shifting effect of the sensor offset due to the reproducibility lack of gas sensors (i.e., drift effect) is effectively eliminated without complex digital signal calibrations. In this work, the proposed readout circuit operation and its performances were verified in by simulation using spectre in cadence simulation software. It was implemented on a printed circuit board (PCB) with discrete components to confirm its effectiveness with existing gas sensor systems and commercial viability.

The rest of this paper is organized as follows: Section 2 analyzes the characteristics of the gas sensor signals by power spectral density measurements. Section 3 describes the proposed readout circuit structure with the offset tracking technique. The measurement results and discussions about the proposed readout circuit are presented in Section 4, followed by the conclusion in Section 5.

## 2. Analysis of Output Characteristics of Sensor

In this section, we focus on the signal characterization of the slow responsive gas sensor and derive an optimal sensing method, which can be directly applied to existing commercial gas sensors.

**Figure 1:**
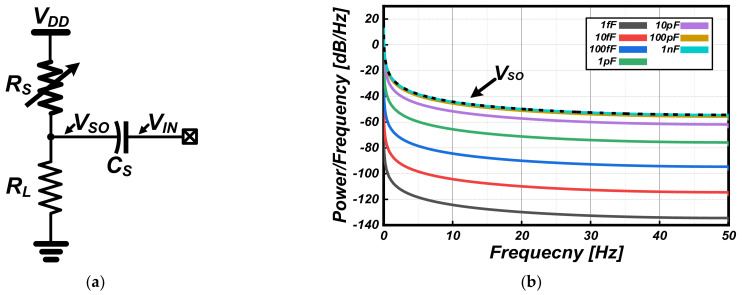
(**a**) Conventional resistive-divider sensing structure with an AC-coupling capacitor. (**b**) Power spectral density of the gas sensor outputs according to the capacitance of C_S_.

Figure 1a shows the conventional resistive-divider sensing structure with an AC-coupling capacitor (C_S_). R_S_ represents the ohmic characteristic of the gas sensor and R_L_ represents the load resistor to define the bias current. In commercial products, this structure is mostly utilized as the basic gas sensor readout circuit for resistance-to-voltage (RV) conversion, which permits only the amount of change in the gas sensor to be transmitted to the readout circuit through the C_S_. Note that the gas sensor has a different offset value for each sample. Based on the published data sheet information for the Figaro TGS2600 [20,21] sensor, in the case of slow response gas sensing, it is assumed that the R_S_ is varied from 100 to 1 kΩ depending on gas concentration and that the R_L_ is 100 kΩ under a supply voltage (V_DD_) of 3.3 V. Figure 1b shows the power spectral density (PSD) of the gas sensor outputs (V_SO_ and V_IN_) according to the capacitance of C_S_. For the condition of the same sensing time (equal to settling time of the R_S_), the signal power of the V_IN_ varies according to the capacitance of C_S_. Note that V_IN_ is the input signal to the gas sensor that is actually read out by the next stage readout circuit. It is clear from the PSD results that the low frequency signal power is dominant in the total output signal power of the gas sensors, and that a large C_S_ is needed to extract the amount of change. When the capacitance of C_S_ becomes larger than 1 nF, the PSDs of the V_SO_ and V_IN_ become similar.

**Figure 2:**
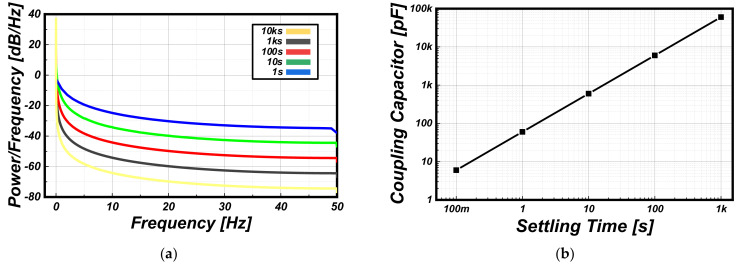
(**a**) PSD results of V_SO_ according to changes in the settling time of R_S_ and (**b**) the capacitance required for V_IN_ settling according to the gas sensor’s sensitivity.

To further investigate the signal characteristics for the condition of the same gas concentration, the PSDs of V_IN_ was analyzed for each sensitivity of the gas sensor. The sensitivity of each gas sensor is modeled with different settling times of R_S_ for the same sensing time. Here, the settling time of R_S_ from 1 to 10,000 s was considered with a C_S_ of 1 pF. Figure 2a shows the PSD results of V_SO_ according to changes in the settling time of R_S_. With a constant capacitance of C_S_, which is to be expected, the proportion of signal power for each frequency decreases as the required settling time for R_S_ increases. This implies that a suitable capacitance of C_S_ should be considered for accurate signal transmission, depending on the sensitivity of the target gas sensor. In Figure 2b, for obtaining the signal transmission over than 3-sigma (3δ) within the sensing time of 1 s, the required capacitance of C_S_ is shown according to the settling time of R_S_. Note that the sensitivity of each sensor was modeled in proportion to the settling time. When the settling time of R_S_ is relatively decreased by 10 times, the size of C_S_ should be increased approximately 10 times to readout the amount of change of the R_S_ with 3δ accuracy. This indicates that the size of C_S_ should be increased when using a gas sensor with a low sensitivity and slow response characteristics. However, even with the optimized capacitance C_S_ for each gas sensor, the sensing results might be different each time due to the variation of the sensitivity and reactivity according to changes in the ambient conditions.

In term of commercialization, the increasing capacitor size is disadvantageous in price competitiveness. Moreover, it is difficult to utilize different capacitance sizes that depend on the target ambient conditions. For all these reasons, it is worthwhile to study the slow-response gas-sensing readout circuit structure without using a large size capacitance to eliminate the gas sensor offset.

## 3. Proposed Readout Circuit Structure and Its Operation

As discussed in the previous section, for gas sensors that respond slowly to ambient target gases, it is inefficient to apply the correlated-double-sampling (CDS) techniques as in [18,19], which utilize an AC-coupling capacitor in readout circuit input networks to remove the gas sensor offset. In general, commercial gas sensors have different requirements for their optimal performances, which are not optimized for a specific readout circuit configuration and operation. Thus, we focus on a commercially viable readout circuit structure that can effectively obtain the measured information of gases with low reactivity and sensitivity characteristics, while maintaining compatibility with the gas sensor itself.

**Figure 3:**
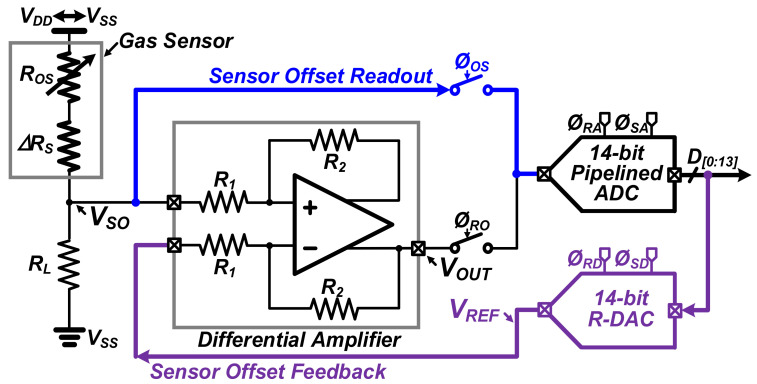
Proposed readout circuit structure for alleviating the sensor offset variation.

Figure 3 presents the proposed readout circuit structure for alleviating the sensor offset variation, which consists of a differential amplifier, a 14-bit analog-to-digital converter (ADC), and a 14-bit DAC. Compared to the conventional readout circuit, instead of an additional reference-generating circuit [12,13,14], the reference-generating DAC has two readout paths (sensor offset readout and feedback). To verify the performance according to the change of the gas sensor offset, the gas sensor was modeled by dividing it into the sensing part (ΔR_S_) and the offset part (R_OS_). In addition, to obtain accurate simulation results, the real signal waveforms measured by the Figaro TGS2600 sensor were used as ΔR_S_. Matching actual design specifications, the differential amplifier was designed with an open loop gain of 95 dB, a common mode rejection rate of 85 dB, a power supply rejection rate of 80 dB, and a slew rate of 160 MHz. Here, R_L_, R_1_, and R_2_ were set as ~10 kΩ, ~50 kΩ, and ~50 kΩ, respectively. For resetting the gas sensor, the supply voltage (V_DD_) for the voltage-driving of the gas sensor can be changed to the ground voltage (V_SS_).

**Figure 4:**
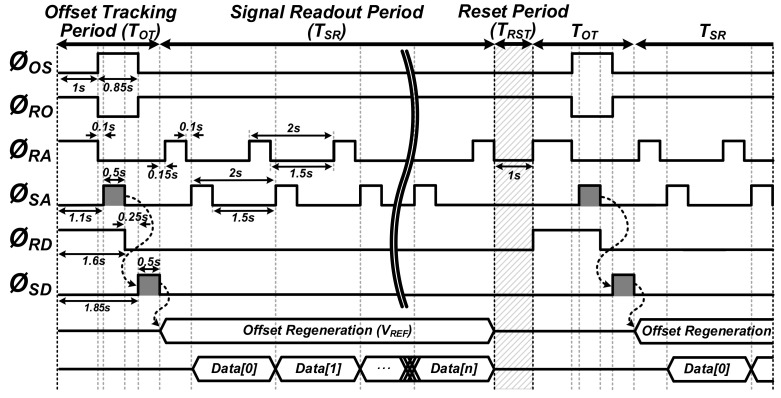
Operational timing diagram of the proposed readout circuit with three phases of offset tracking (T_OT_), signal readout (T_SR_), and sensor reset (T_RST_).

The functional timing diagram of the proposed readout circuit is illustrated in Figure 4, with three phases of offset tracking (T_OT_), signal readout (T_SR_), and sensor reset (T_RST_). First, the offset tracking is performed during T_OT_, to define the output common voltage of V_SO_. After Ø_OS_ is on, the ADC starts to read V_OS_ during Ø_SA_, and its results (D_INIT_) are transferred to the DAC. Then, when Ø_SD_ is on, the DAC regenerates V_REF_ corresponding to the D_INIT_, which is maintained until just before T_RST_. During T_SR_, the differential amplifier performs the operation of subtracting V_REF_ from V_SO_, and the ADC converts it into digital codes (D_SIG_) with Ø_RA_ and Ø_SA_. The output result is only the amount of change of V_SO_. Finally, during T_RST_, the reset operation is performed to initialize the gas sensor itself. In this way, the gas sensor offset can be updated periodically, and even non-periodically, when replacing the gas sensor.

**Figure 5:**
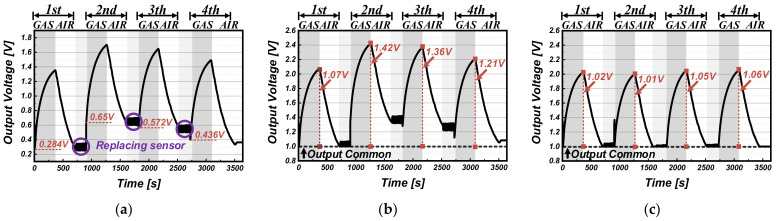
(**a**) Real signal waveform with changes in the sensor offset upwards; simulation results of the (**b**) conventional readout circuit and the (**c**) proposed readout circuit for that input.

**Figure 6:**
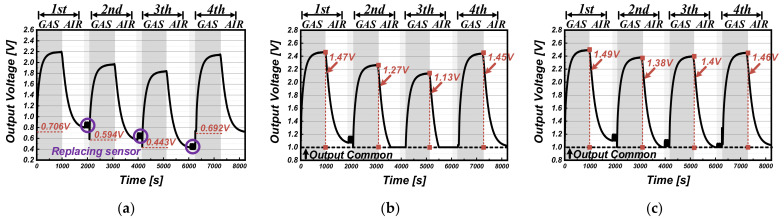
(**a**) Real signal waveform with changes in the sensor offset downwards; simulation results of the (**b**) conventional readout circuit and the (**c**) proposed readout circuit for that input.

The simulation results of the conventional and proposed readout circuit are shown in Figure 5 and Figure 6 with the real signal waveforms in the two cases: the case where the sensor offset changes upwards (Figure 5a) and the case where the sensor offset changes downwards (Figure 6a). Using the Figaro TGS2600 sensor, the gas-sensing results were obtained four times with alternately repeating injection of ethanol (C_2_H_5_OH) and fresh air. The different gas sensor offsets according to ambient condition changes were generated by replacing the sensor samples each sensing time. Table 1 shows the information about the real signal waveforms with the mean sensor resistance R_S_ (= ΔR_S_ + R_OS_). The resistance value of the gas sensor was estimated by the equation of R_S_ ≈ ((V_DD_ − V_SO_)/V_SO_) × R_L_. For the conventional readout circuit (representing in Figure 5b), whole gas-sensing periods from 1st to 4th, only the offset value of the first gas sensor is used as V_REF_ to remove the gas sensor offset; the offsets (0.65 V for the 2nd offset change, 0.572 V for the 3rd offset change, and 0.436 V for the 4th offset change) are larger than the first defined V_REF_ of 0.284 V (from the 1st sensor offset). Because the conventional readout circuit extracts just difference between V_SO_ and V_REF_, the offset variation is not properly removed (offset error) and rather is amplified as the output signal, which exacerbates the non-uniformity in measuring the same gas concentration. On the other hand, as shown in Figure 6b, when the sensor offsets (0.594 V for 2nd offset change, 0.443 V for 3rd offset change, and 0.692 V for 4th offset change) are lower than the first defined V_REF_ of 0.706 V (from 1st sensor offset), the output signal is clamped by the offset error, resulting in degradation of the sensing accuracy. These errors are because the conventional readout circuit does not reflect the change in the initial value of the input signal at all.

However, the proposed readout circuit updates V_REF_ for every sensing measurement by tracking the initial value of V_SO_. Sequentially, in Figure 5c, V_REF_ becomes 0.284 V, 0.65 V, 0.572 V, and 0.436 V, and in Figure 6c, 0.706 V, 0.594 V, 0.443 V, and 0.692 V. As a result, regardless of the offset variation of gas sensors, the proposed readout circuit extracts only amount of change in the slow-responsive gas, while maintaining a uniform reactivity at the corresponding gas concentration.

In this study, we adopt the differential amplifier to extract the changed amount of the gas sensor, because it is advantageous to evaluate the performance of the proposed readout structure under various test environments. Since the amplifier performances are an important factor for determining signal-to-noise ratio (SNR) for readout structures, the readout structure with improved performances could be developed if further research is carried out on the amplifier circuitry.

## 4. Measurement Results and Discussion

**Figure 7:**
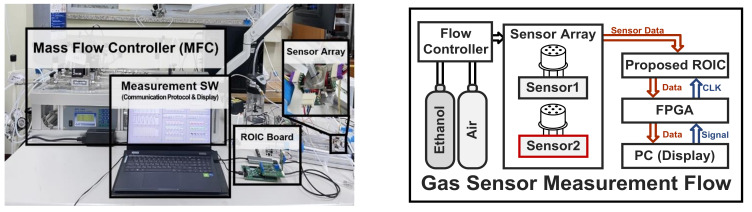
Gas-sensing measurement environment and gas sensor measurement flow.

Figure 7 shows the measurement environment for ethanol (C_2_H_5_OH) gas sensing that consists of a mass flow controller (MFC, Line Tech M3030V) for controlling the flow rate of C_2_H_5_OH gas and fresh air, a commercially available gas sensor array (the Figaro TGS2600), an evaluation board with the proposed readout circuit, and a measurement software program. The flow rate was controlled to 400 sccm for C_2_H_5_OH gas and 300 sccm for fresh air through the MFC. In addition, the injection time of C_2_H_5_OH gas and fresh gas was set to be from a minimum of 300 s to a maximum of 900 s. Because the target ethanol gas is continuously injected into the sensor array under the MFC, there is no attenuation of the target gas concentration.

**Figure 8:**
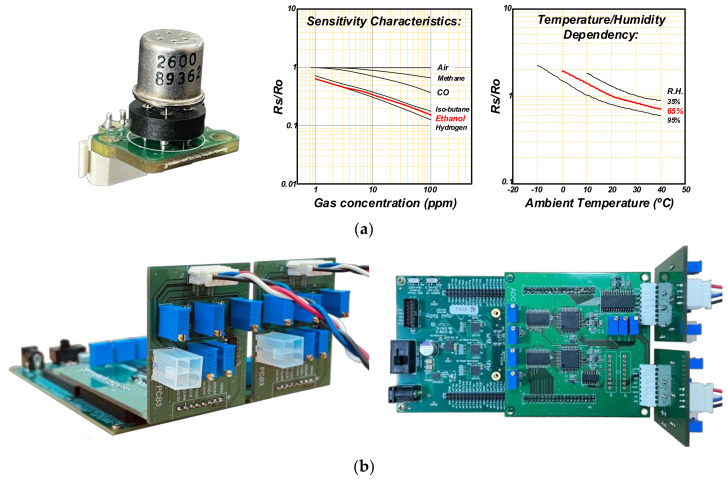
(**a**) Gas sensor board with Figaro TGS2600 and (**b**) the evaluation board with the proposed readout circuit.

In this work, as shown in Figure 8a, the Figaro TGS2600 sensor was used for sensing C_2_H_5_OH gas at a concentration of ~50 ppm while considering its technical specifications. All gas-sensing measurements were conducted at normal temperature (20 ± 2 °C) and relative humidity is R.H. 65 ± 5% in fresh air. Figure 8b shows the evaluation board assembly that consists of the readout circuit board and commercial FPGA (XEM-7305 [22]) board. The readout circuit board has two gas-sensing channels to compare the performance of the conventional and proposed readout circuit structure. For further evaluation in various measurement environments, the control signals and operating timing are generated through the external FPGA mounted on the evaluation board. To verify compatibility and operation in terms of commercialization, in this work, the proposed readout structure was implemented on a PCB board using general-purpose discrete elements of differential amplifier, ADC, and DAC, to verify its effectiveness in various commercial gas-sensing systems. Thus, to enhance power efficiency, 1 kSPS ADC/DAC was used where the ADC runs once every 100 Hz frequency for sensing the changes in the gas sensor. In order to consider bit resolution, since the noise performance of commercial gas sensors (Figaro TGS2600) was not disclosed, a low-noise differential amplifier and 14-bit noise-guaranteed ADC/DAC were used so that the noise limitation would not occur in the differential amplifier and ADC/DAC. Then, we observed the signal output, including noises of the gas sensor itself. Here, we chose the differential amplifier with noise performance of 16 $\mathrm{nV}/\sqrt{\mathrm{Hz}}$ at 1 MHz bandwidth and the 14-bit ADC/DAC with input referred noise of 0.36 LSB_rms_ at the full reference scale of 2.5 V. In addition, to consider noise bandwidth, an additional external capacitor was added to the output node of the differential amplifier, resulting in a reduction of the noise bandwidth to ~10 kHz. This can be further optimized when the proposed readout structure is developed into an integrated circuit. The output codes from the proposed readout circuit were transmitted to the PC through the USB interface and processed using a custom MATLAB software program to display the real-time imaging on the screen. The capturing software program with various verification functions was customized using MATLAB programming.

**Figure 9:**
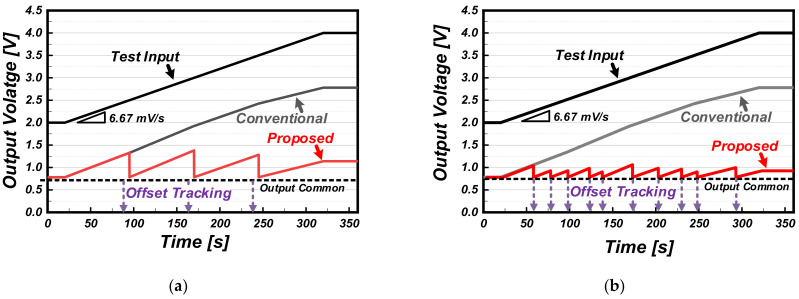
Measured output waveforms of conventional and proposed readout circuit under the condition of a linearly increasing sensor offset: (**a**) periodic offset tracking and (**b**) aperiodic offset tracking results.

Figure 9 shows the output waveforms of the prototype readout circuit in which the measured ADC outputs are expressed as an analog output voltage. The offset tracking operation of the prototype readout circuit was verified using a ramping test input so that the gas sensor signal would be constant, but its offset increased linearly due to changes in the ambient environment. Here, a ramp signal with the slope of 6.67 mV/s was used. Compared to the conventional readout circuit, as shown in Figure 9a, the proposed readout circuit extracts the amount of change in the test input based on the initial value updated at each periodic offset tracking time. In addition, as shown in Figure 9b, it can be operated non-periodically according to any changes in conditions. The allowable offset tracking range is limited by the output dynamic range of the reference generating DAC.

**Figure 10:**
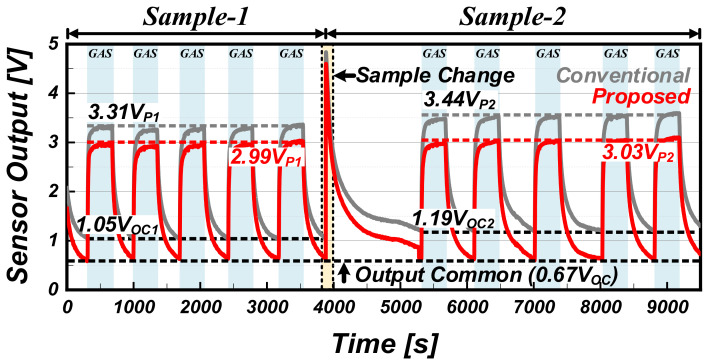
Measurement results of the conventional and proposed readout circuit for two commercial gas sensors.

Figure 10 shows the measurement results of the conventional readout circuit (representing the gray colored cells) and the proposed readout circuit (representing red colored) for two TGS2600 samples (Samples 1 and 2) with similar sensitivities but different initial values. To continuously compare the two sensors under the same test conditions, the gas sensor sample was replaced during the measurement (at 3800 s). For a gas concentration of 50 ppm, from the conventional readout circuit, Sample 1 exhibits the output voltage of 3.31 V_P1_ at the initial value of 1.05 V_OC1_, whereas Sample 2 exhibits the output voltage of 3.44 V_P2_ at the initial value of 1.19 V_OC2_. Here, the average of the initial and output values of the sensing signal was used as a representative value. Owing to the difference in the initial value between the gas sensor samples, different sensor outputs are extracted even at the same gas concentration. From the perspective of a sensor system, this degrades the discrimination against each gas concentration changes, causing gas detection non-uniformity. On the contrary, in the case of the proposed readout circuit, the initial value of both samples is set to 0.67 V_OC_, and based on this, Sample 1 and Sample 2 extract output voltages of 2.99 V_P1_ and 3.03 V_P2_, respectively. Considering the difference in sensitivity of the gas sensor sample itself, for the same gas concentration, these results are similar output values by minimizing the initial value difference.

**Figure 11:**
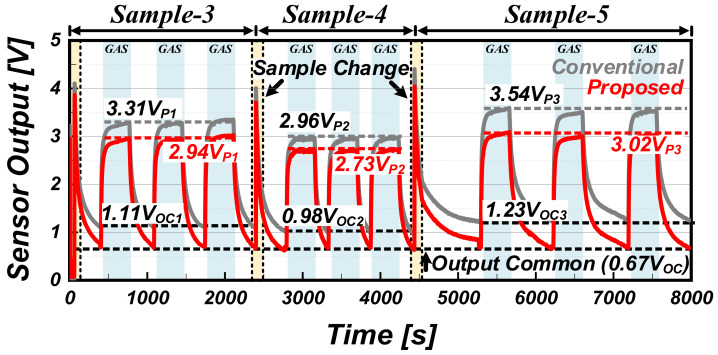
Measurement results for various gas sensor samples with various sensitivities and initial values.

Figure 11 shows the measurement results for various gas sensor samples (Samples 3, 4, and 5) with various sensitivities and initial values. In general, gas sensors have different initial values and different sensitivities for each gas sensor, so it is difficult to extract the uniform output value corresponding to an accurate gas concentration without a complicated post-processing process. However, the proposed readout circuit minimizes the difference in the initial value for each gas sensor, and it is effective even in compensating the difference in sensitivity for each gas sensor. Note that, for sensitivity compensation, the initial value of the gas sensor should be set to a constant value. Table 2 and Table 3 list information on the measured sensor resistance per measurement time used in Figure 10 and Figure 11, respectively.

It is meaningful to discuss how to accurately extract the signals of the gas sensors without the proposed offset tracking technique. Once the signals of the gas sensors are converted into digital information, a reference digital value should be defined for the digital signal to be meaningful information. However, considering the sensitivity of the gas sensor to the surrounding environment, it is not easy to determine a specific reference value that covers all measurement situations. As an example of digital processing, the output change from the gas sensor is recognized as a sensing signal by classifying digital signals through pattern recognition and defining minimum and maximum values. If this digital algorithm is implemented as digital logic or through post-processing, it is disadvantageous in terms of price competitiveness due to increased area occupation and power consumption. However, the proposed readout structure is relatively cost-effective to extract the amount of change from the gas sensor because it does not require any complex digital circuitry.

## 5. Conclusions

This paper presents a commercially viable readout circuit structure targeting slow response gas sensors. In this paper, the optimized readout circuit structure for slow-reacting gas signals was verified in the simulation. It was then implemented on a PCB board to verify its effectiveness in various commercial gas-sensing systems. While maintaining the optimal operating condition of the gas sensor, regardless of the initial value of the gas sensor, the proposed readout circuit can effectively extract a uniform gas-sensing output for each gas concentration. In addition, the proposed design does not require large capacitors to extract the measurements, which will make the IC design more cost-effective and flexible. Although this study did not develop an integrated circuit (IC), the principle was proven using discrete components and the design could be the basis for developing an IC.

## Figures and Tables

**Table 1:** Information regarding the real signal waveforms with the mean sensor resistance.

Input Condition	Ethanol Concentration [ppm]	Figure 5a		Figure 6a	
		Measurement Time [s]	Mean Sensor Resistance [kΩ]	Measurement Time [s]	Mean Sensor Resistance [kΩ]
Ethanol	50	0~360	166.06~26.93	0~950	60.82~12.83
Air	0	360~720	26.93~154.47	950~1900	12.83~50.98
Replacing Sensor	0	720~900	X	1900~2080	X
Ethanol	50	900~1260	62.46~19.43	2080~3030	74.18~15.35
Air	0	1260~1620	19.43~70.39	3030~3980	15.35~77.57
Replacing Sensor	0	1620~1800	X	3980~4160	X
Ethanol	50	1800~2160	77.41~20.49	4160~5110	102.87~17.17
Air	0	2160~2520	20.49~79.77	5110~6060	17.17~100.62
Replacing Sensor	0	2520~2700	X	6060~6260	X
Ethanol	50	2700~3060	104.68~23.6	6260~7210	62.25~13.26
Air	0	3060~3600	23.6~127.74	7210~8200	13.26~58.97

**Table 2:** Information regarding the measured sensor resistance per measurement time in Figure 10.

Input Condition	Ethanol Concentration [ppm]	Sensor 1		Sensor 2	
		Measurement Time [s]	Mean Sensor Resistance [kΩ]	Measurement Time [s]	Mean Sensor Resistance [kΩ]
Ethanol	50	310~670	109.4~15.31	5310~5670	93.96~13.06
Air	0	670~1000	15.31~110.18	5670~6110	13.06~95
Ethanol	0	1000~1360	110.18~16.04	6110~6470	95~12.5
Air	50	1360~1690	16.04~110.58	6470~7010	12.5~95.52
Ethanol	0	1690~2050	110.58~16.32	7010~7370	95.52~12.54
Air	0	2050~2420	16.32~108.88	7370~8030	12.54~92.54
Ethanol	50	2420~2780	108.88~15.59	8030~8390	92.54~12.38
Air	0	2780~3180	15.59~111.11	8390~8810	12.38~94.79
Ethanol	50	3180~3540	111.11~14.77	8810~9170	94.79~11.84
Air	0	3540~3880	14.77~115.91	9170~9700	11.84~102.97
Replacing Sensor	X	3880~5310	X	X	X

**Table 3:** Information regarding the measured sensor resistance per measurement time in Figure 11.

Input Condition	Ethanol Concentration [ppm]	Sensor 3		Sensor 4		Sensor 5	
		Measurement Time [s]	Mean Sensor Resistance [kΩ]	Measurement Time [s]	Mean Sensor Resistance [kΩ]	Measurement Time [s]	Mean Sensor Resistance [kΩ]
Ethanol	50	410~770	104.89~15.81	2790~3150	120.6~20.74	5300~5660	93.45~12.24
Air	0	770~1090	15.81~105.74	3150~3340	20.74~121.36	5660~6240	12.24~89.8
Ethanol	50	1090~1450	105.74~16.21	3340~3700	121.36~20.65	6240~6600	89.8~12.78
Air	0	1450~1740	16.21~104.77	3700~3870	20.65~120.75	6240~7190	12.78~89.6
Ethanol	50	1740~2100	104.77~15.28	3870~4230	120.75~20.47	7190~7550	89.6~12.46
Air	0	2100~2400	15.28~107.99	4230~4440	20.47~125.11	7550~8000	12.46~89.71
Replacing Sensor	X	2400~2790	X	4440~5300	X	X	X

## Data Availability

Not applicable.
